# Chromium-Modified Ultrathin CoFe LDH as High-Efficiency Electrode for Hydrogen Evolution Reaction

**DOI:** 10.3390/nano12071227

**Published:** 2022-04-06

**Authors:** Jun-Jun Zhang, Meng-Yang Li, Xiang Li, Wei-Wei Bao, Chang-Qing Jin, Xiao-Hua Feng, Ge Liu, Chun-Ming Yang, Nan-Nan Zhang

**Affiliations:** 1 Shaanxi Key Laboratory of Optoelectronic Functional Materials and Devices, School of Materials Science and Chemical Engineering, Xi’an Technological University, Xi’an 710021, China; limengyang1997lmy@163.com (M.-Y.L.); lixiang@xatu.edu.cn (X.L.); eaglejin@xatu.edu.cn (C.-Q.J.); fxh1232022@163.com (X.-H.F.); L1462310794@126.com (G.L.); 2 National & Local Joint Engineering Laboratory for Slag Comprehensive Utilization and Environmental Technology, School of Material Science and Engineering, Shaanxi University of Technology, Hanzhong 723000, China; baowei1834@163.com; 3 Shaanxi Key Laboratory of Chemical Reaction Engineering, College of Chemistry & Chemical Engineering, Yan’an University, Yan’an 716000, China; chunmingyang@yau.edu.cn; 4 Instrumental Analysis Center, Shanghai Jiao Tong University, Shanghai 200240, China; zhangnn19@sjtu.edu.cn

**Keywords:** chromium-modified, hydrogen evolution reaction, free-standing, layered double hydroxides, electron transfer

## Abstract

Hydrogen evolution reaction (HER) has a dominant function in energy conversion and storage because it supplies a most effective way for converting electricity into sustainable high-purity hydrogen. Layered double hydroxides (LDHs) have shown promising performance in the process of electrochemical water oxidation (a half-reaction for water splitting). Nevertheless, HER properties have not been well released due to the structural characteristics of related materials. Herein, a simple and scalable tactics is developed to synthesize chromium-doped CoFe LDH (CoFeCr LDH). Thanks to oxygen vacancy, optimized electronic structure and interconnected array hierarchical structure, our developed ternary CoFeCr-based layered double hydroxide catalysts can provide 10 mA cm^−2^ current density at −0.201 V vs. RHE with superior long-term stability in alkaline electrolyte. We anticipate that the simple but feasible polymetallic electronic modulation strategy can strengthen the electrocatalytic property of the layered double hydroxides established in the present study, based on a carbon neutral and hydrogen economy.

## 1. Introduction

By increasing the proportion of green energy used and reducing the use of non-renewable energy, the carbon neutral process can be made to happen better [1]. The electrolysis of water (utilizing electric energy to drive hydrogen production without CO_2_ emissions) can more effectively work out the balance for environmental pollution and energy shortages. Now, the electric propulsion water decomposition needs high-cost electrodes to overcome adverse energy barriers of the involved reaction process [2]. The most efficient electrode for HER is still noble metal Pt or Pt/C. Nevertheless, the high expense and insufficient reserves of Pt limit its utility in industrial scale [3]. 

In the past decades, substantial effort and research have been devoted in the preparation of high-efficiency transition metal and carbon-based electrocatalysts [4]. Among various electrocatalysts, LDHs in particular have attracted much attention on account of their 2D layered structure, interlayer spacing, tunable electronic structure and metal species, which could simultaneously increase the number of active sites and expedite electron transport [5]. Introducing heteroatoms or other composite systems to increase the activity of the electrode, from the point of view of surface and/or interface, is a common strategy [6]. The electron density in the metal center of the composite system can be optimized by introducing high valence metal ions [7]. Chen et al. have developed FeCoNi(S) oxygen evolution and overall water splitting catalysts and suggested that reconstructed Ni(Fe,Co)OOH is the real active sites [8]. Fu’s group synthesized MoSe_2_-based HER electrodes catalysts with a more complex piezo-flexoelectric coupling effect at 1T and 2H-phase interfacial areas by etching Mo metallic mesh through an optimized hydrothermal route [9]. Yang Shao-Horn and colleagues reported a new type of metal hydroxide-organic frameworks synthesized through combining aromatic carboxylate linkers on the LDHs. The results showed that excellent performance comes from optimized Ni redox and the binding of intermediates [10]. More importantly, the most promising strategy is to maximize catalytic sites utilization and improve the synergy of mass and electron transport [11,12]. Hence, it is a key challenge to fabricate highly efficient and inexpensive catalysts with open architecture to better balance electron transport and mass transfer [13].

To address the above problems, in this research, hierarchical chromium-modified cobalt iron layered double hydroxides electrodes were systematically fabricated by means of a one-step electrodeposition strategy. Such an integrated CoFeCr LDH nanostructure remarkably improves the ability of catalytic process. More importantly, −0.201 V vs. RHE is needed to achieve 10 mA cm^−2^ over the optimized CoFeCr LDH with oxygen vacancy electrocatalyst. The remarkable performance and long-term stability of CoFeCr LDH were attributed to the synergy effect between structural and electronic modulation. In particular, this research is conducive to better understanding the polymetallic modulation based on advanced LDHs catalyst for energy conversion.

## 2. Materials and Methods

### 2.1. Materials

Cobalt (II) nitrate hexahydrate (AR) and Chromic nitrate nonahydrate (AR) were purchased from DAMAO. Platinum carbon (Pt/C, 20 wt%), ammonium hydroxide (AR) and potassium hydroxide (AR) were obtained from INNOCHEM, MACKLIN and CMACKLIN KESHI, respectively. Ultrapure water was used throughout the experiment process.

### 2.2. Preparation of CoFeCr LDH and CoFe LDH Electrodes

The electrodes were prepared by electrodeposition process. The whole electrodeposition was carried out by using CHI660E workstation, and saturated calomel, Pt mesh and beforehand Ni foam served as reference electrode, counter electrode and work electrode, respectively. The composition of electrodeposition solution changed with the chromium doping concentration while the moles of cobalt ions and total moles of metal ions remained the same. The specific composition of the electrodeposition solution is shown in Table S1. Take the 10% CoFeCr LDH for example; the aqueous solution (40 mL) contained 2.5 mmol Co(NO_3_)_2_·6H_2_O, 0.875 mmol FeCl_2_·4H_2_O and 0.375 mmol Cr(NO_3_)_3_·9H_2_O was firstly sonicated for 15 min to accelerate dissolution and then 0.37 mL NH_3_·H_2_O was added. The electrodeposition process was operated at specific potential (−2.0 V vs. SCE) [14]. The deposition times were set as 60, 80, 100, 200 and 300 s. After the electrochemical test, the sample with 100 s (10%-CoFeCr-LDH-100s) had best performance for HER and its loading was ~0.4 mg cm^−2^. The catalyst load was determined by weight change before and after electrodeposition process.

### 2.3. Characterizations of CoFeCr LDH and CoFe LDH Electrodes

The SEM measurements were performed on a scanning electron microscope (FESEM, JSM-7610F, 10 kV). The TEM and HR-TEM measurements were taken with a JEOL JEM-F200 microscope. The samples were prepared by dropping ethanol dispersion of samples onto carbon-coated copper TEM grids using pipettes and dried under ambient condition. The X-ray photoelectron spectroscopy (XPS) measurements were conducted on a Kratos Axis Ultra DLD spectrometer. The electron paramagnetic resonance (EPR) spectroscopy was probed by a Bruker E580 spectrometer at room temperature (295 K).

### 2.4. Electrochemical Measurements

All electrochemical tests were performed in a conventional three-electrodes system by the CHI660E workstation (CHI Instruments, Shanghai, China) at room temperature with the as-prepared electrodes (1 cm∗1 cm) as work electrodes, carbon rod as counter electrode, Hg/HgO as reference electrode and 1.0 M KOH as electrolyte. All linear sweep voltammetry (LSV) curves towards HER in this work were conducted at a scan rate of 10 mV s^−^^1^ and calibrated by *iR* corrected. Electrochemical impedance spectroscopy (EIS) was measured with AC impedance over a frequency range from 0.1 to 10^5^ Hz. The potentials used in this work were calibrated to RHE by the equation: E (vs. RHE) = E (vs. Hg/HgO) + 0.098 + 0.059 pH.

## 3. Results

**Figure 1:**
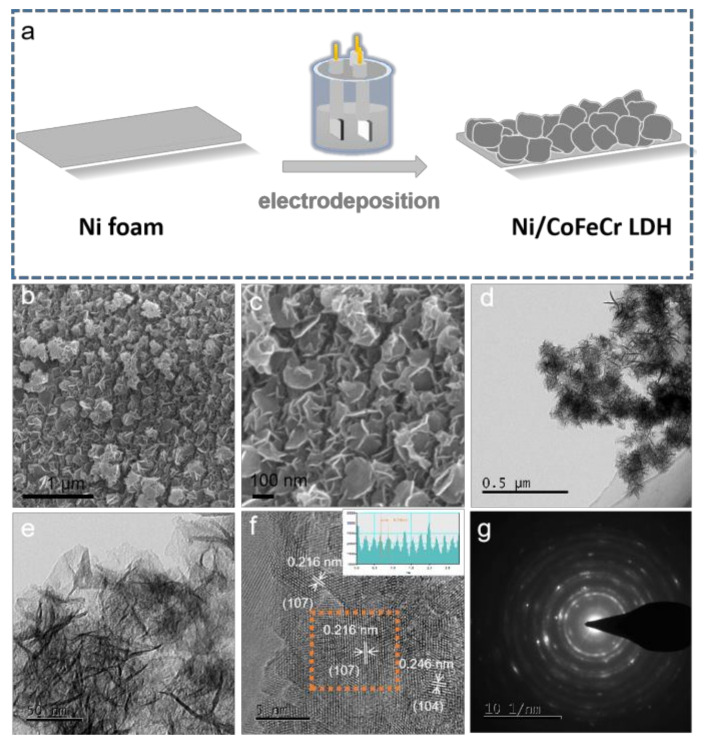
(**a**) Schematic diagram of sample preparation, (**b**,**c**) scanning electron microscope images, (**d**,**e**) transmission electron microscope images, (**f**) high resolution transmission electron microscopy image and (**g**) selected area electron diffraction pattern of the CoFeCr LDH electrocatalysts.

In this paper we developed an electrodeposition process to synthesize chromium-modified cobalt iron layered double hydroxides directly grown on Ni foam in a general solution (Co, Fe, Cr metal ions) based on optimized concentration and time, as illustrated in Figure 1a. The detailed synthesis parameters and process are provided in the electronic Supplementary Material (Table S1). Commercial nickel was selected as the substrate due to low cost and multi-dimensional characteristic. Mass transport and electron transport are two major regulatory directions of electrochemistry. The best efficiency can be achieved only by maintaining a balance between the mass transport and electron transport. For the nickel foam, due to its open structure, the catalytic dose can be increased to a certain extent without affecting electron transport. However, when the catalyst is overloaded, electron transport is affected [15]. The microscopic morphology images Figure 1b,c and Figure S1 show that the prepared material adhered well to the surface of the Ni foam. A further observation declares that the as-fabricated CoFeCr LDH catalysts consist of a large number of nanoscale lamellar structures. The stability of the whole array structure can be improved by cross-linking between the nanosheets [16].

**Figure 2:**
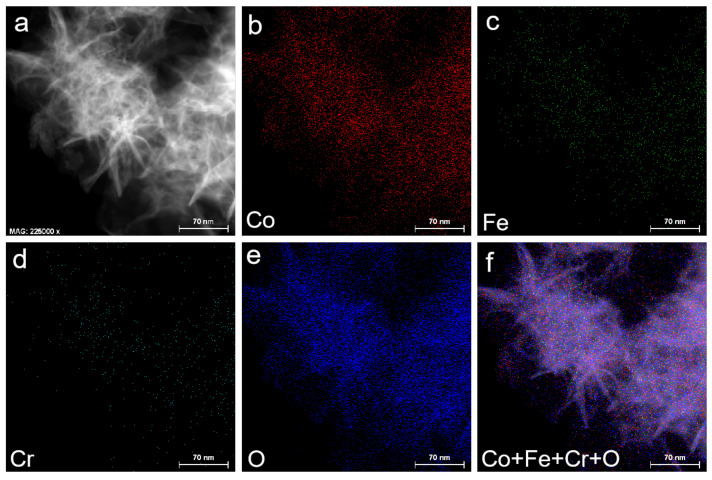
The corresponding elements mapping of CoFeCr LDH nanosheets array electrocatalyst. (**a**) STEM image of CoFeCr LDH. (**b**–**f**) EDX elemental mapping spectra of Co, Fe, Cr, O and mixed, respectively.

To gain the results of structure and chemical composition, the CoFeCr LDH electrode was investigated by transmission electron microscope (TEM) and selected area electron diffraction (SAED) element mapping, respectively. The TEM results displayed that the nanosheets were assembled together with high uniformity (Figure 1d,e). The interplanar separation in the HR-TEM image (Figure 1f) was calculated to be 0.216 and 0.246 nm, corresponding to (107) and (104) planes of trimetallic CoFeCr layer double hydroxides, respectively [17]. The inset of Figure 1f shows that the spacing is determined to be 0.216 nm. The SAED image of CoFeCr LDH sample further confirms the good crystallinity and polycrystalline property (Figure 1g and Figure S2) [18]. The energy-dispersive X-ray (EDX) spectrum displays the existence of Co, Fe, Cr and O elements (Figure S3). Figure 2 shows elemental mapping images, which demonstrate that the four elements (Co, Fe, Cr and O) are evenly distributed across the whole CoFeCr LDH nanosheets, and no partial aggregation of chromium can be observed.

**Figure 3:**
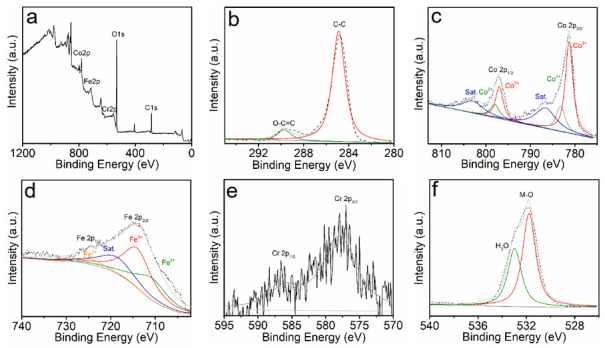
The XPS spectra of the CoFeCr LDH catalysts. (**a**) Survey spectra, (**b**) C1s, (**c**) Co 2p, (**d**) Fe 2p, (**e**) Cr 2p and (**f**) O 1s.

The surface chemical compositions and oxidation states of elements in CoFeCr LDH samples were investigated by X-ray photoelectron spectroscopy (XPS). The CoFeCr LDH survey spectra demonstrated the presence of Co, Fe, Cr, C and O elements (Figure 3a). The C1s spectrum of CoFeCr LDH shows two board peaks (284.9 eV and 289.7 eV), corresponding to C-C and O-C=C (Figure 3b), respectively [19]. The Co 2p spectrum of CoFeCr LDH includes four obvious Co peaks (Figure 3c), in which the peaks (781.2 eV) and (797.0 eV) correspond to the Co^3+^ species, and the peaks (783.2 eV) and (798.1 eV) belong to the Co^2+^ species. The Fe 2p spectra shown in Figure 3d also exhibits the presence of oxidation state of Fe^2+^ (710.2 eV) and Fe^3+^ (714.8 eV), respectively [20]. The Cr 2p spectrum (Figure 3e) of CoFeCr LDH electrode shows two board peaks at 586.7 eV (Cr 2p_1/2_) and 578.0 eV Cr 2p_3/2_ (Cr^3+^) [21]. Simultaneously, two peaks of O 1s spectrum at 531.7 and 533. 0 eV correspond with M-O and H_2_O, respectively [22]. The above characterization well indicates that we have prepared a polymetallic hydroxides CoFeCr LDH sample.

**Figure 4:**
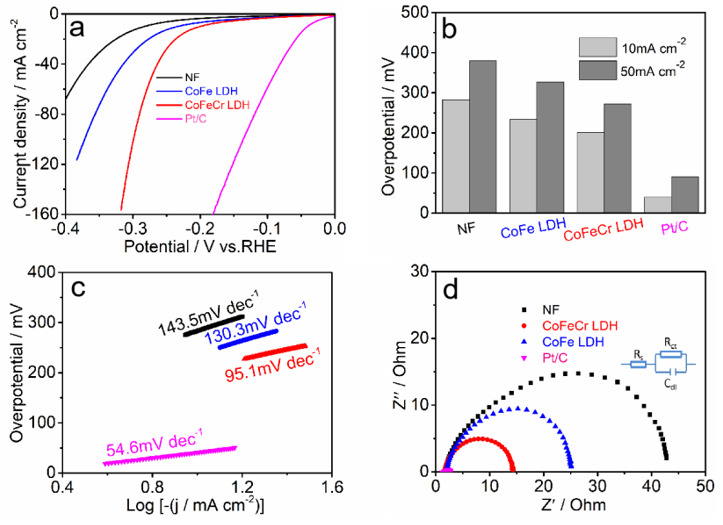
(**a**) The *iR*-corrected polarization curves, (**b**) a comparison of the corresponding overpotentials at 10 mA cm^−2^ and 50 mA cm^−2^ current outputs, (**c**) corresponding Tafel plot and (**d**) the EIS and equivalent circuit model (insert) of the CoFe LDH, CoFeCr LDH, Pt/C and bare NF electrodes.

We first assessed the properties of the chromium-modified cobalt iron layered double hydroxides array for HER. The HER performance of the CoFeCr LDH and other reference catalysts (CoFe LDH, Pt/C and Ni substrate) was evaluated by utilizing a standard three-electrodes system in 1 M KOH solution. The CoFeCr LDH electrode was directly used as the working electrode. The CoFeCr LDH exhibited a low onset potential. As shown in Figure 4a, the prepared electrode revealed excellent performance towards HER (201 mV overpotential at 10 mA cm^−2^, 265 mV overpotential at 50 mA cm^−2^) as compared with CoFe LDH electrode (Figure 4a,b). Although there is still room for improvement compared with Pt/C (39 mV overpotential at the current density 10 mA cm^−2^), our catalysts showed a better advantage when compared with related non-precious metals, such as NiFe-LDH/FeCoS_2_/CFC (380 mV at 10 mA cm^−2^), Ni_1−x_Fe_x_^−^LDH (242 mV at 10 mA cm^−2^), CoFe LDH^−^F (255 mV at 10 mA cm^−2^) and CoMoV LDH/NF (270 mV at 10 mA cm^−2^), as listed in Table S2 (see comparison details) [23,24,25,26].

The catalytic kinetics of the CoFeCr LDH electrode and other reference electrodes was analyzed by the Tafel slopes and the electrochemical impedance spectroscopy. With a small Tafel slope, the hydrogen evolution rate would increase rapidly with less increment of the overpotential. The CoFeCr LDH electrode reveals a smaller Tafel slope of 95.1 mV dec^−1^ compared with that of CoFe LDH (130.3 mV dec^−1^), indicating superior HER kinetics (Figure 4c) [27]. The electrochemical impedance spectroscopy curves also illustrate smaller electron transfer resistance (R_ct_) and thus faster HER kinetics for the CoFeCr LDH electrode, comparing with the undoped CoFe LDH electrode (Figure 4d) [28].

**Figure 5:**
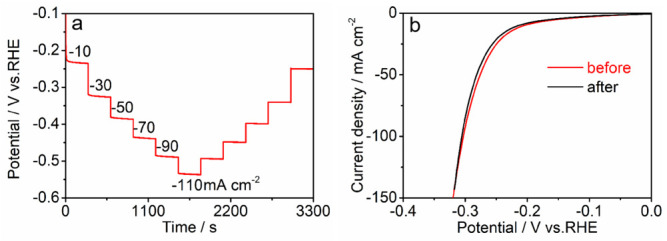
(**a**) The multi-current steps and (**b**) iR-corrected polarization curves of the electrode before and after 500 cycles (Scanned area: −1.1~1.5 V vs. Hg/HgO, Potential scan rate: 200 mV s^−^^1^).

**Figure 6:**
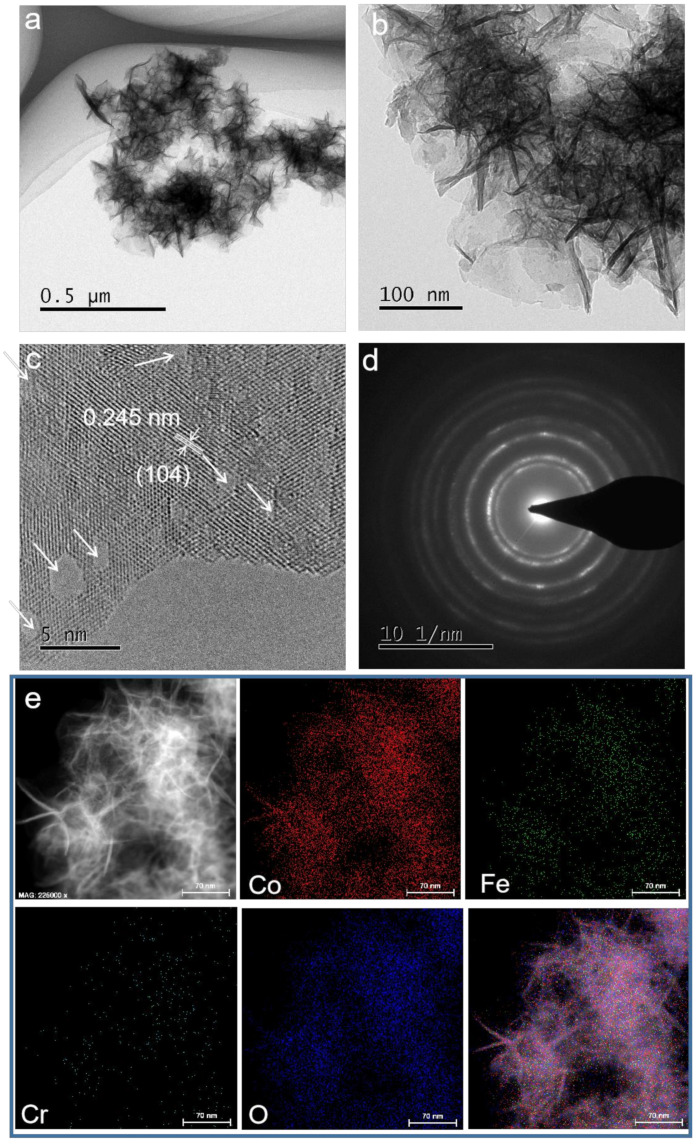
(**a**,**b**) Transmission electron microscopy images, (**c**) high resolution transmission electron microscopy image, (**d**) selected area electron diffraction pattern and (**e**) the corresponding element mapping of the CoFeCr LDH catalysts after the stability test.

The long-term durability is another investigation objectives for practical electrochemical process [29]. Figure 5a shows that the as-prepared CoFeCr LDH electrode can be keep stable under a variety of current output. In the meantime, the HER durability of the CoFeCr LDH electrode was evaluated by performing cyclic voltammetry process. After 500 cycles (Figure 5b), the potential of a given current (50 mA cm^−2^) has increased by only 5.7 mV. No identified degradation was discovered after 24 h continuous working under a constant current density, revealing that the combined electrode possesses excellent long-term stability (Figure S4). The CoFeCr LDH electrodes that operate for a long time were further characterized by several advanced tests. Figure S5 presents the SEM image of the used CoFeCr LDH electrode, revealing the nanosheets morphology and array architecture. The result is consistent with the pristine CoFeCr LDH electrode (Figure 1). At the same time, the stability of the two-dimensional structure was also proved by TEM results (Figure 6a,b). The results of HR-TEM showed that there were more nanopores in the nanosheet structure and more defect sites could be increased in the catalytic process (Figure 6c and Figure S7). After a long period of operation, the electrode still retains the polycrystalline nature and LDHs phase structure (Figure 6c,d). The EDX result displays the existence four metal elements (Co, Fe, Cr, O) in the used CoFeCr LDH electrode (Figure S6). Figure 6e shows elemental mapping images, which demonstrate that the elements are evenly distributed across the whole CoFeCr LDH nanosheets.

The valence changes of elements were investigated by XPS test. As the result shows, the peak positions of each element basically remain unchanged, indicating that the catalytic electrode has excellent stability (Figure S8a,b). Nonetheless, compared with the initial CoFeCr LDH electrode, the total content of Cr is reduced during the HER process. The EPR test at room temperature shown in Figure S9 illustrates that the defect degree of CoFeCr LDH remained during the long-term electrolysis [30]. A variety of characterization techniques have demonstrated that the CoFeCr LDH catalyst has high stability. We also studied the stability of hydrogen evolution in acid electrolyte. The results show that the electrode also has moderate stability under acidic conditions (Figure S10), and further research is in progress.

**Figure 7:**
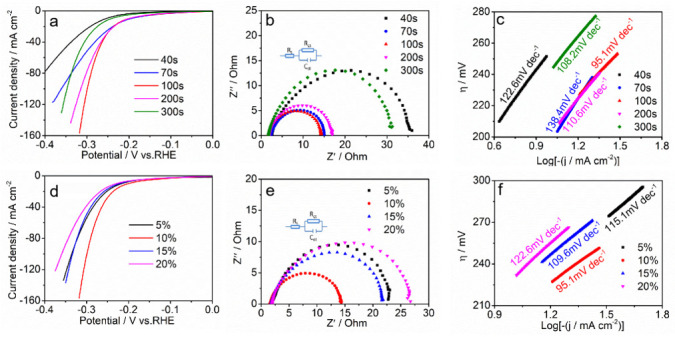
(**a**,**d**) The *iR*-corrected polarization curves, (**b**,**e**) the EIS spectra and equivalent circuit model (insert) and (**c**,**f**) corresponding Tafel plot of CoFeCr LDH electrodes with different deposition time and doping concentrations, respectively.

We performed a series of optimizations for the parameters involved in the catalyst preparation process. The deposition time affects the catalyst load. The right amount of catalyst has an important effect on both electron transport and electron transport. By comparing samples with different times, we confirm that the best deposition time is 100 s (Figure 7a), and the best catalyst has high catalytic activity and fast electron transfer Figure 7b,c and Figure S11). Furthermore, we optimized the doping concentration of Cr species. When the doping concentration is 10% (Figure 7d), the electrode has the highest catalytic performance due to the lowest transfer resistance (Figure 7e,f and Figure S12).

## 4. Discussion

The activity improvement of the CoFeCr LDH electrode was discussed in detail. To understand the surface chemical state of the as-prepared electrodes, X-ray photoelectron spectroscopy (XPS) measurement was conducted. Compared with the CoFe LDH sample, an additional faint Cr element peak was found in the survey spectra of the CoFeCr LDH, demonstrating the successful doping of the Cr species into CoFe LDH substrate. It is worth noting that both peaks (Co 2p_3/2_ and Co 2p_1/2_) shift towards lower energy (0.15 eV) due to doped Cr, verifying the synergistic electronic interactions of metal ions among Co, Fe and Cr cations [30,31,32,33]. The introduced Cr element has a noticeable influence on the chemical microenvironment of the CoFe LDH electrode. In Cr 2p spectra (Figure 2d), the two deconvoluted peaks (586.7 eV and 578 eV) should be attributed to Cr 2p_1/2_ and Cr 2p_3/2_ (Cr^3+^) (Figure 3e). At the same time, we found that the position of Fe 2p peak did not change significantly after Cr doped. The phenomenon indicates that the introduction of foreign Cr causes the selective change of metals electron density in the CoFeCr LDH catalyst. The change of electron density can promote electrochemical adsorption and desorption [30,34,35].

**Figure 8:**
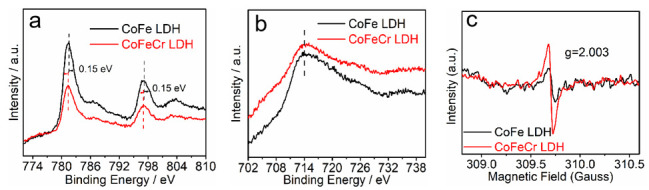
X-ray photoelectron spectroscopy (**a**) Co 2p, (**b**) Fe 2p and (**c**) the electron paramagnetic resonance spectra of CoFeCr LDH and CoFe LDH electrodes.

To further verify the existence of the oxygen vacancies, room temperature electron paramagnetic resonance (EPR) was performed. The evident signal peak was at g = 2.003, which is identified as the essential feature of surface oxygen vacancy [36,37]. As shown in Figure 8c, after the introduction of foreign Cr element, the signal strength representing oxygen defects was enhanced. The presence of oxygen deficiency can increase the electron transport in catalytic process, thus enhancing the adsorption/desorption of related intermediates [38,39]. Combined with our previous published article [20], we believe that Ni foam as a substrate also plays an indispensable role in improving the performance of the catalysts. The tight interface between the Ni foam substrate and catalyst layer can regulate the electron density of the metal and accelerate the electron transfer process (Figure S13). In addition to the improvement of electron transfer, the enhancement of mass transfer by array structure also plays a pivotal role in improving the overall efficacy.

## 5. Conclusions

In this work, we designed a CoFeCr LDH hybrid for water splitting through one-step electrodeposition strategy. The prepared electrode shows high performance in hydrogen production, only requiring 201 mV overpotential to drive current output (10 mA cm^−2^) with an outstanding stability in 1.0 M KOH electrolyte. The CoFeCr LDH HER electrode is distinctly more active than the previous reported oxyhydroxide HER catalysts. The work demonstrates the effect of electrode composition on improving the intrinsic properties and optimizing the electronic structure of electrode. Considering the fine control of this technique to high quality water splitting catalysts, the work emphasizes a competitive strategy to obtain cheap HER layered double hydroxides electrodes.

## Data Availability

Data sharing is not applicable to this article as no new data were created or analysed in this study.

## Supplementary Materials

The following supporting information can be downloaded at: https://www.mdpi.com/article/10.3390/nano12071227/s1, Figure S1: The SEM of CoFeCr LDH electrode; Figure S2: The HRTEM and corresponding fast-Fourier-transform (FFT) of the as-obtained CoFeCr LDH electrode; Figure S3: The EDX of CoFeCr LDH electrode; Figure S4: The chronopotentiometry measurement of CoFeCr LDH electrode; Figure S5: The SEM images of CoFeCr LDH electrode after long-time stability test; Figure S6: The EDX of CoFeCr LDH electrode after long- time stability test; Figure S7: The HRTEM image of CoFeCr LDH electrode after long- time stability test; Figure S8: High-resolution XPS (a) Co 2p, (b) Fe 2p and (c) Cr 2p spectra of CoFeCr LDH electrode before and after long- time stability test; Figure S9: The electron paramagnetic resonance (EPR ) spectra of CoFeCr LDH electrode before and after long time stability test; Figure S10: (a) the polarization curves of the electrode before and after 500 cycles. (b) the multi-potential steps and (c) the multi-current steps; Figure S11: The comparison of the corresponding overpotentials at 10 mA cm^−2^ and 50 mA cm^−2^ current outputs based on different deposition time electrodes; Figure S12: The comparison of the corresponding overpotentials at 10 mA cm^−2^ and 50 mA cm^−2^ current outputs based on different doping concentrations electrodes; Figure S13: The high-resolution (a) Co 2p and (b) Fe 2p spectra of CoFe LDH electrode and CoFe LDH powder; Table S1: Summary of parameters for sample preparation; Table S2: Comparison of the HER performance of CoFeCr LDH catalyst with other reported OER catalysts. References [40,41,42,43,44,45,46,47,48,49,50] are cited in the Supplementary Materials.
