# Supplementary material for: Chromium-Modified Ultrathin CoFe LDH as High-Efficiency Electrode for Hydrogen Evolution Reaction
Source: Nanomaterials (Basel). 2022 Apr 6;12(7):1227. doi: 10.3390/nano12071227 (PMC9002697; doi:10.3390/nano12071227)
Supplement: Supplementary file 1 [file nanomaterials-12-01227-s001.zip › nanomaterials-1645680-supplementary.pdf]

Supporting Information

Chromium-Modified Ultrathin CoFe LDH as High-Efficiency Electrode for Hydrogen Evolution Reaction

Jun-Jun Zhang [1,*], Meng-Yang Li [1], Xiang Li [1], Wei-Wei Bao [2], Chang-Qing Jin [1], Xiao-Hua Feng [1], Ge Liu [1], Chun-Ming Yang [3] and Nan-Nan Zhang [4]

[1] Shaanxi Key Laboratory of Optoelectronic Functional Materials and Devices, School of Materials Science and Chemical Engineering, Xi'an Technological University, Xi'an 710021, China;
limengyang1997lmy@163.com (M.-Y.L.); lixiang@xatu.edu.cn (X.L.); eaglejin@xatu.edu.cn (C.-Q.J.); fxh1232022@163.com (X.-H.F.); L1462310794@126.com (G.L.)

[2] National & Local Joint Engineering Laboratory for Slag Comprehensive Utilization and Environmental Technology, School of Material Science and Engineering, Shaanxi University of Technology,
Hanzhong 723000, China; baowei1834@163.com

[3] Shaanxi Key Laboratory of Chemical Reaction Engineering, College of Chemistry & Chemical Engineering, Yan'an University , Yan'an 716000, China; chunmingyang@yau.edu.cn

[4] Instrumental Analysis Center, Shanghai Jiao Tong University, Shanghai 200240, China; zhangnn19@sjtu.edu.cn

* Correspondence: zhangjunjun@xatu.edu.cn

List of Contents

1. Experimental Section:

Preparation of Pt/C

2. Supplementary Figures:

Figure S1. The SEM of CoFeCr LDH electrode.

Figure S2. The HRTEM and corresponding fast-Fourier-transform (FFT) of the as-obtained CoFeCr LDH electrode.

Figure S3. The EDX of CoFeCr LDH electrode.

Figure S4. The chronopotentiometry measurement of CoFeCr LDH electrode.

Figure S5. The SEM images of CoFeCr LDH electrode after long-time stability test.

Figure S6. The EDX of CoFeCr LDH electrode after long- time stability test.

Figure S7. The HRTEM image of CoFeCr LDH electrode after long- time stability test.

Figure S8. High-resolution XPS (a) Co 2p, (b) Fe 2p and (c) Cr 2p spectra of CoFeCr LDH electrode before and after long- time stability test.

Figure S9. The electron paramagnetic resonance (EPR) spectra of CoFeCr LDH electrode before and after long time stability test.

Figure S10. (a)the polarization curves of the electrode before and after 500 cycles. (b) the multi-potential steps and (c) the multi-current steps.

Figure S11. The comparison of the corresponding overpotentials at 10 mA cm^{-2} and 50 mA cm^{-2} current outputs based on different deposition time electrodes.

Figure S12. The comparison of the corresponding overpotentials at 10 mA cm^{-2} and 50 mA cm^{-2} current outputs based on different doping concentrations electrodes.

Figure.S13 The high-resolution (a) Co 2p and (b) Fe 2p spectra of CoFe LDH electrode and CoFe LDH powder.

3.Supplementary Tables:

Table S1. Summary of parameters for sample preparation.

Table S2. Comparison of the HER performance of CoFeCr LDH catalyst with other reported OER catalysts

4.Notes and references

1. Experimental details

Preparation of Pt/C

Prior to the synthesis, NFs were sonicated in 3 M HCl, ethanol and ultrapure water for 15min to clean the surface. 1.2mg Pt/C was dispersed into a solution of water, ethanol and Nafion with a volume ratio of 60/120/15 via sonication. Coat a third of the total well-dispersed solution to the cleaned NF surface.

2. Supplementary Figures:

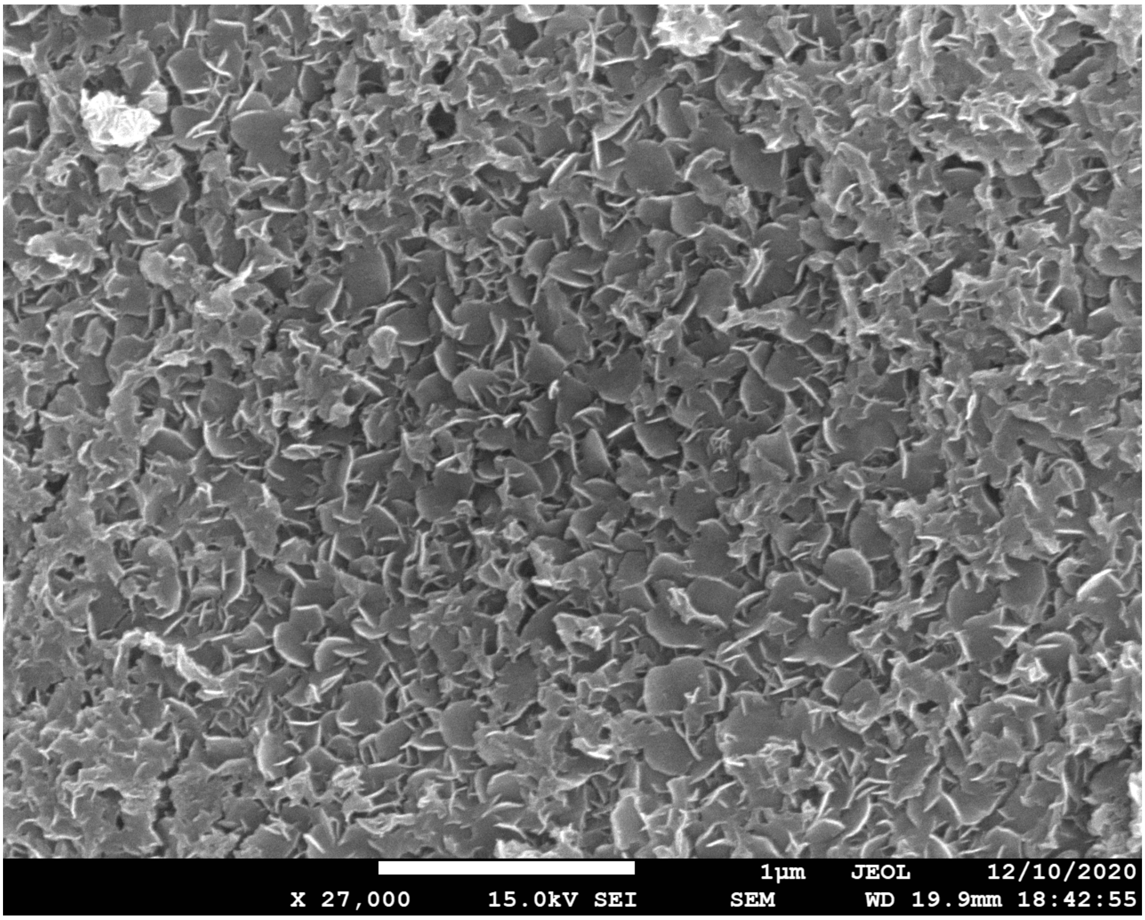

Figure S1. The SEM of CoFeCr LDH electrode. The results show that the electrode has obvious array structure, which is beneficial to mass transfer in the catalytic process.

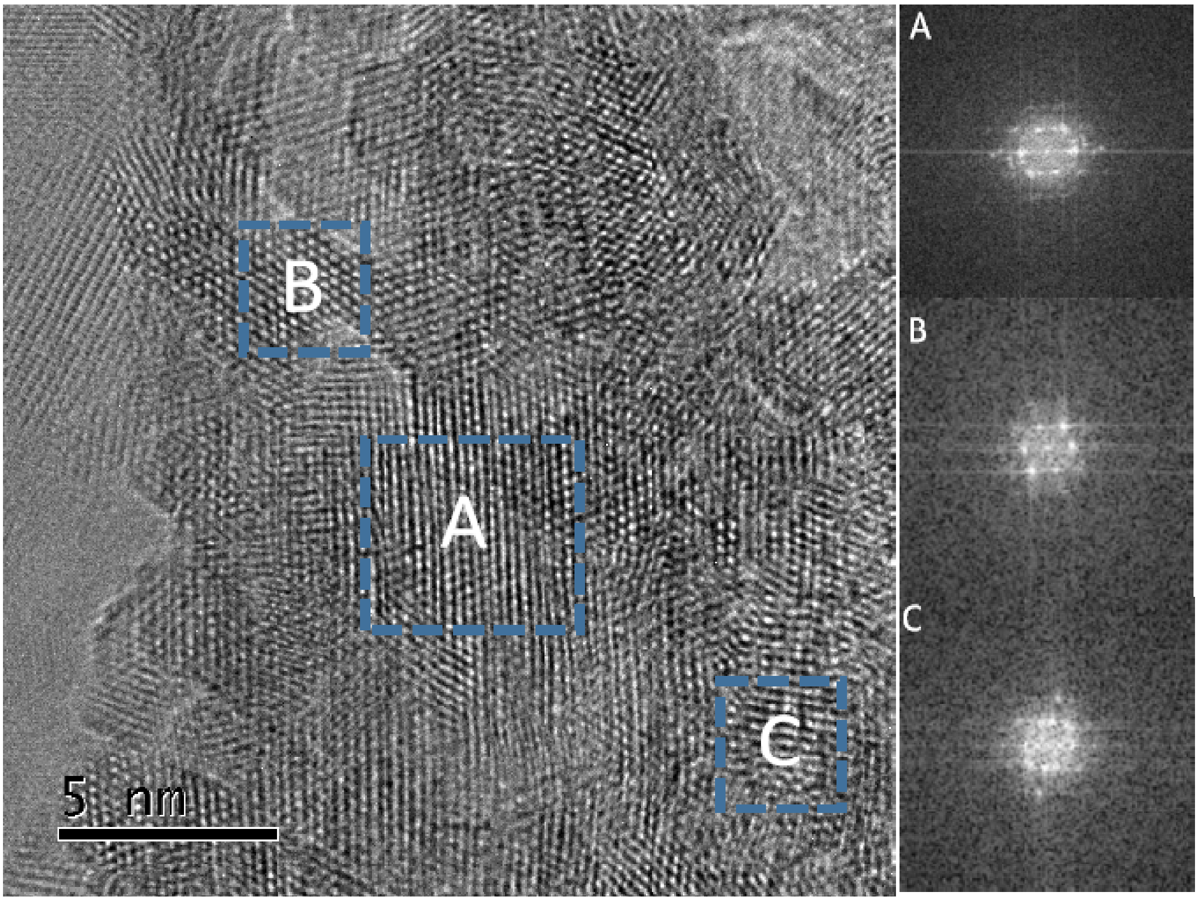

Figure S2. The HRTEM and corresponding fast-Fourier-transform (FFT) of the as-obtained CoFeCr LDH electrode. The results show that the sample is polycrystalline in nature.

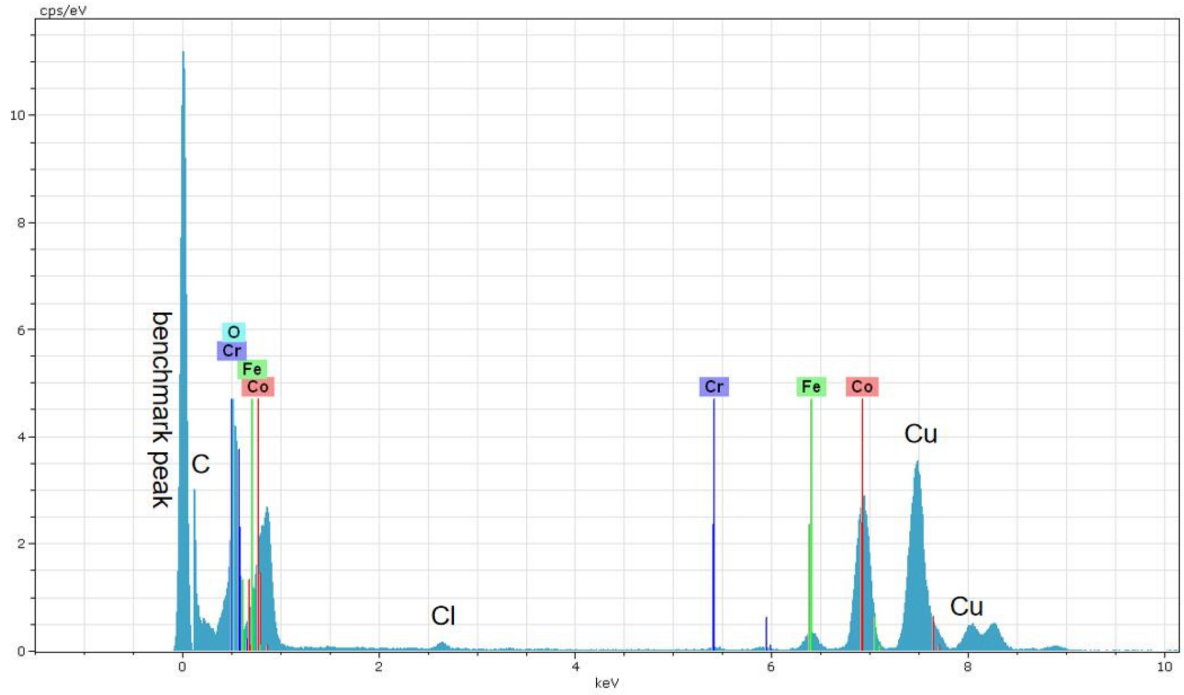

Figure S3. The energy-dispersive X-ray (EDX) of CoFeCr LDH electrode.

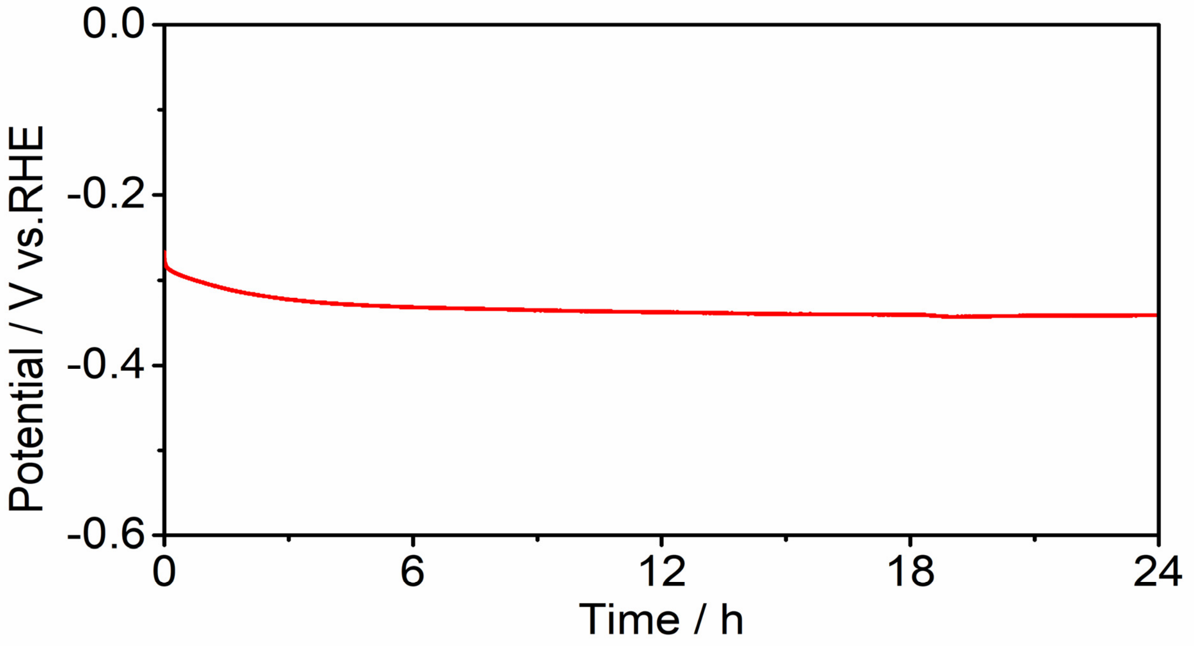

Figure S4. The chronopotentiometry measurement of CoFeCr LDH electrode. The current density used for the chronopotentiometry measurement is 10 mA cm^{-2}. The electrolyte used for the test is 1.0 M KOH.

.

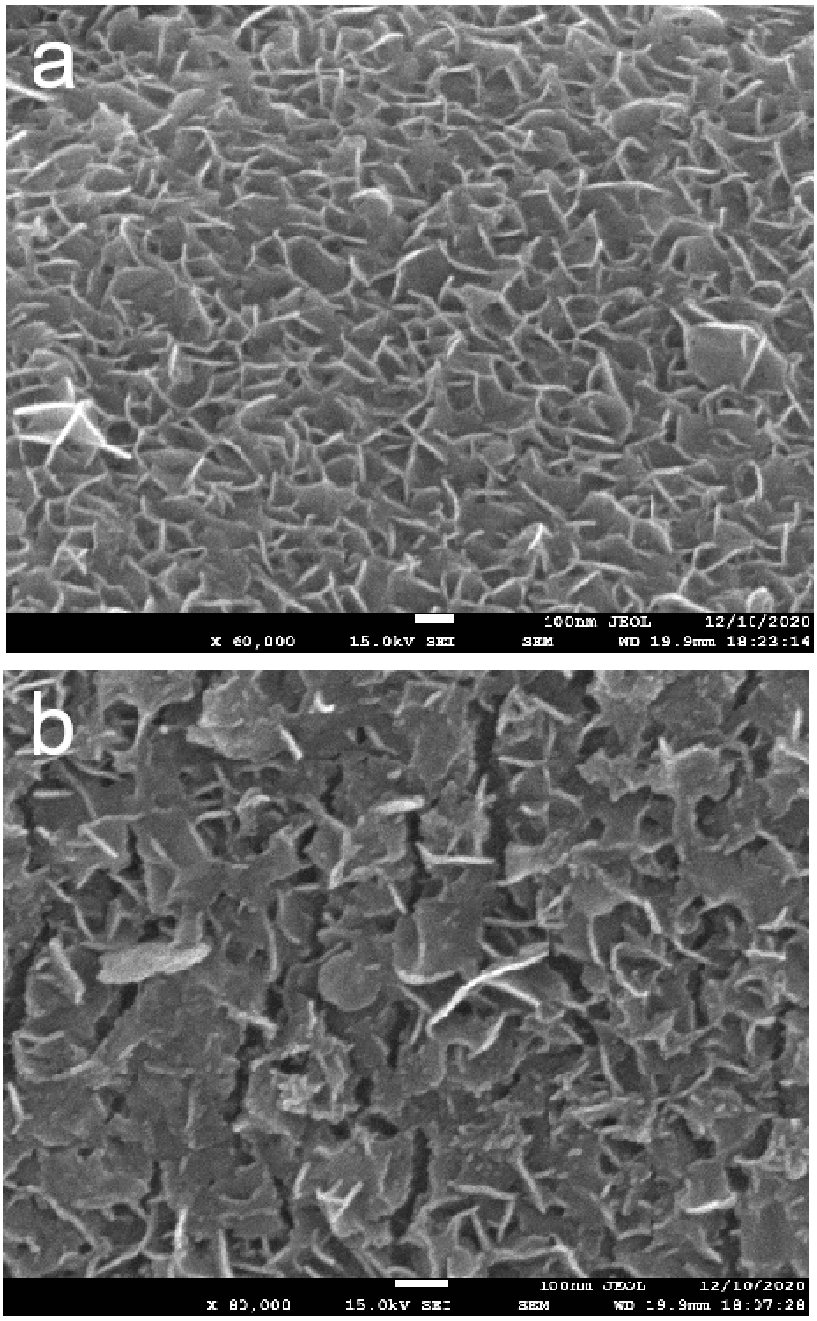

Figure S5. The SEM images of CoFeCr LDH electrode after long-time stability test.

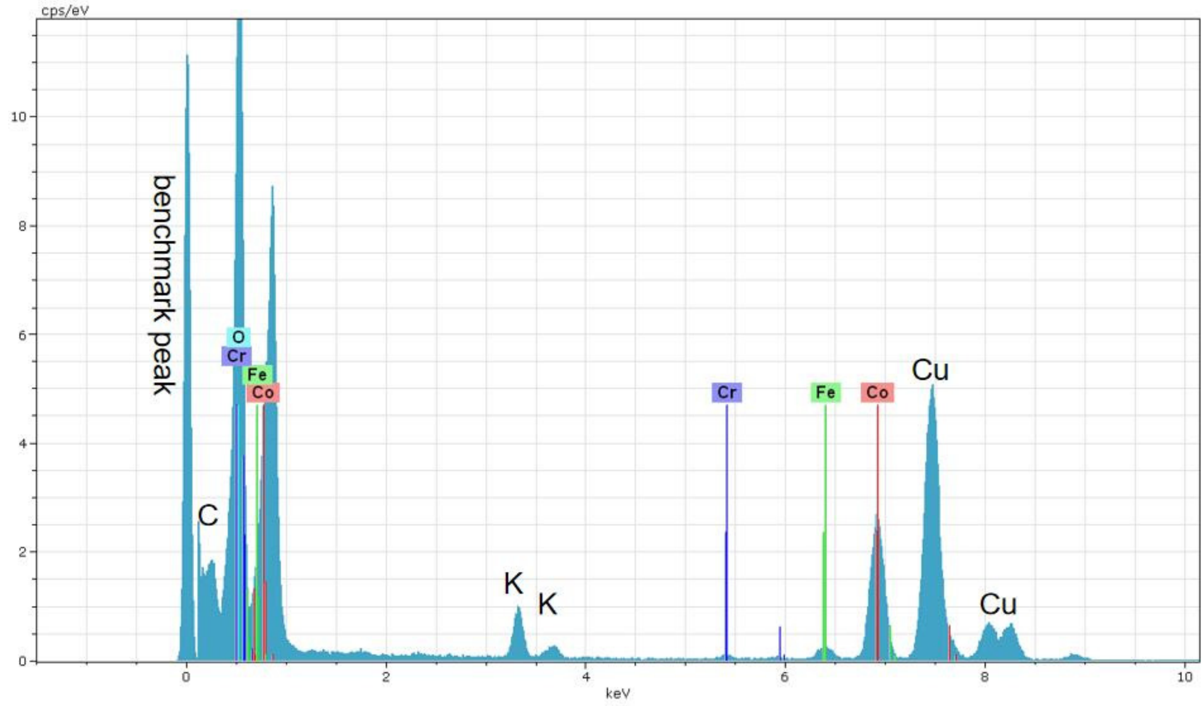

Figure S6. The energy-dispersive X-ray (EDX) of CoFeCr LDH electrode after long-time stability test.

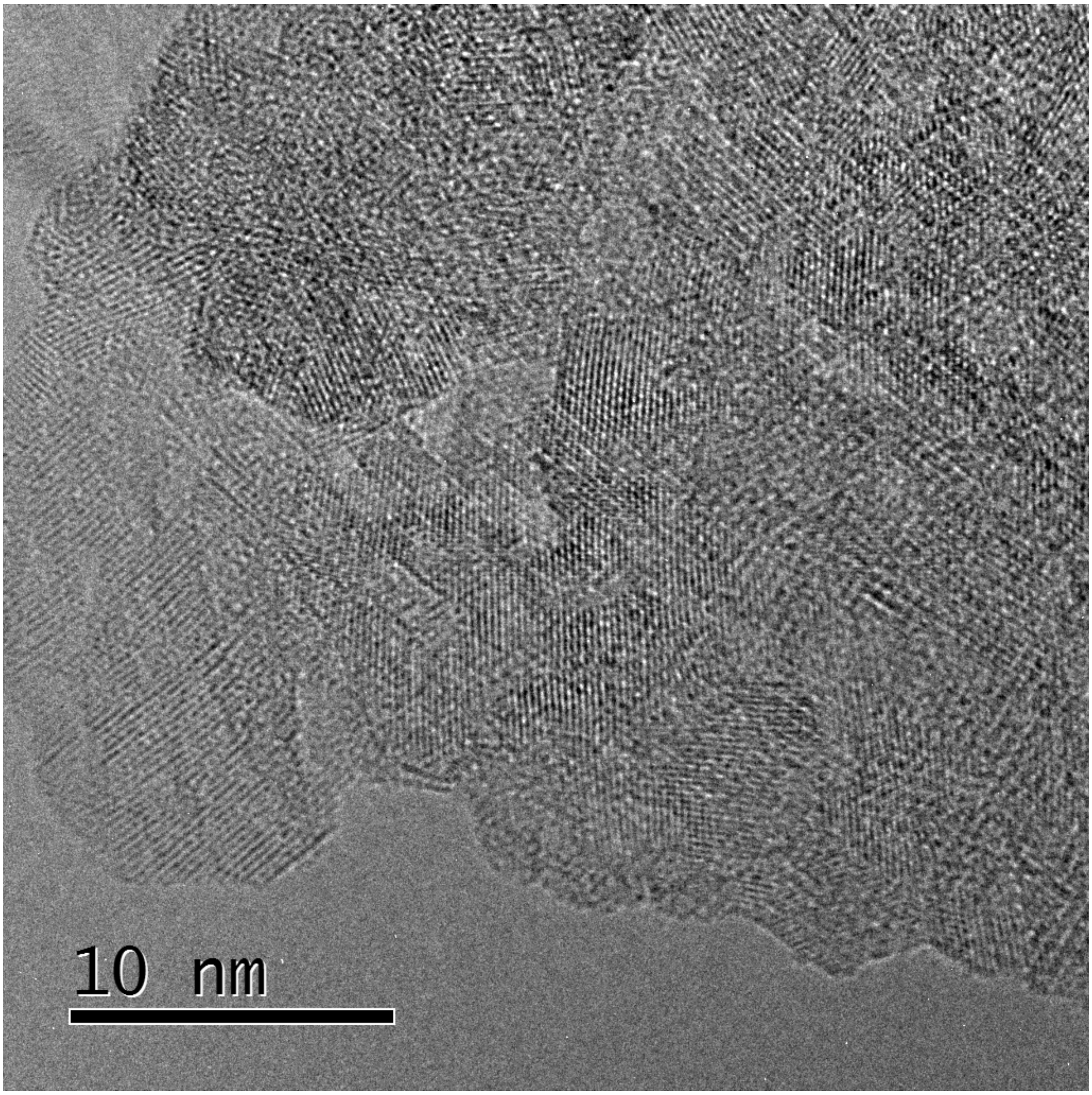

Figure S7. The HRTEM image of CoFeCr LDH electrode after long- time stability test.

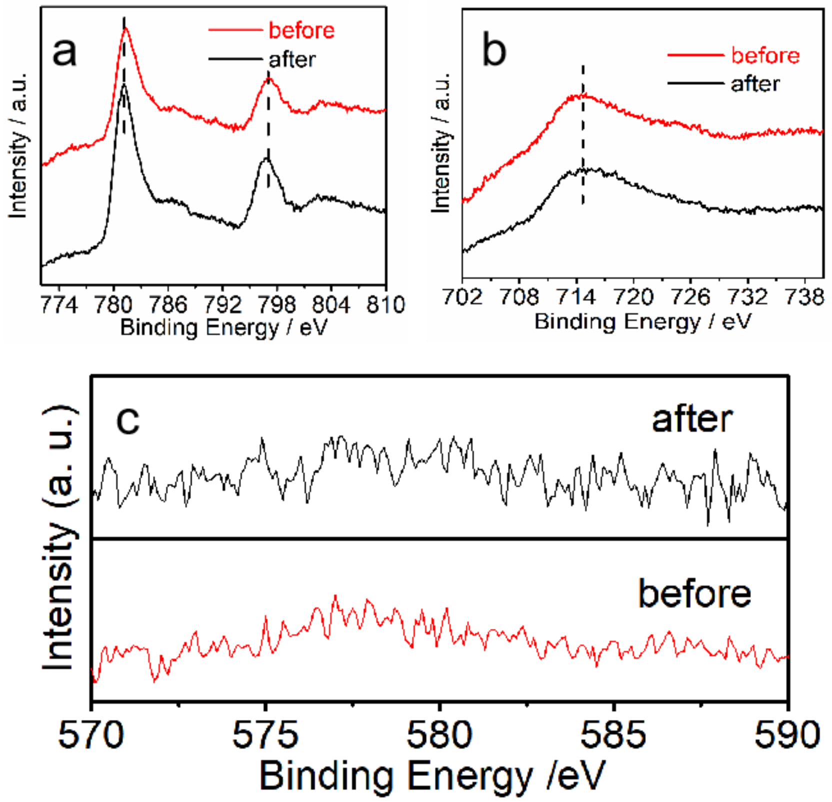

Figure S8. High-resolution XPS (a) Co 2p, (b) Fe 2p and (c) Cr 2p spectra of CoFeCr LDH electrode before and after long-time stability test.

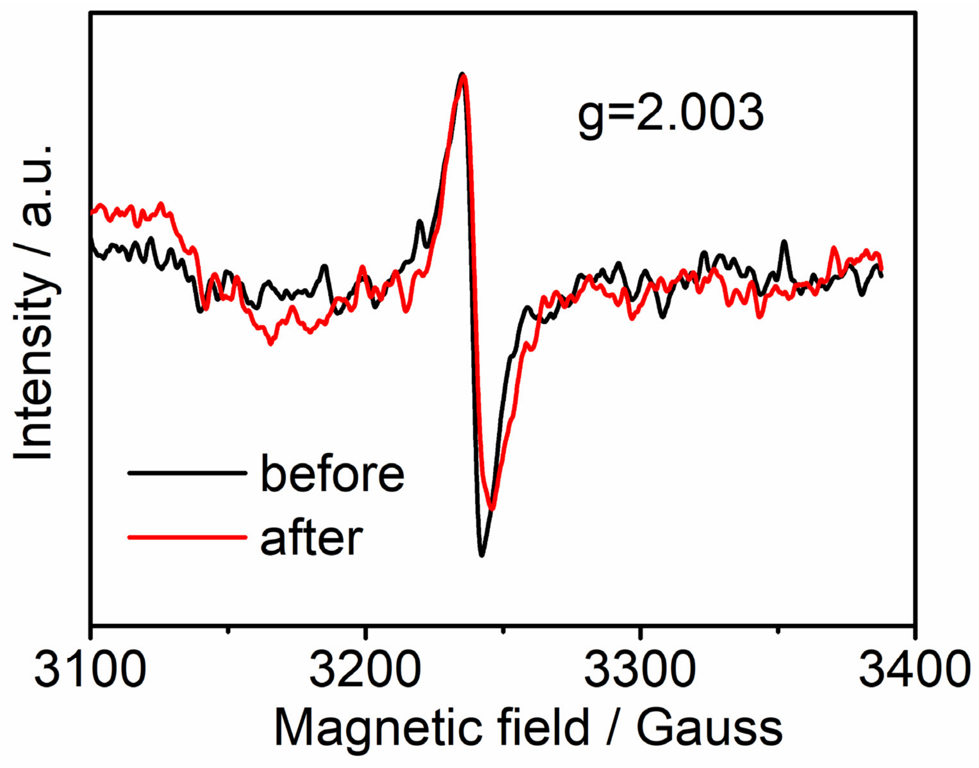

Figure S9. The electron paramagnetic resonance (EPR) spectra of CoFeCr LDH electrode before and after long time stability test.

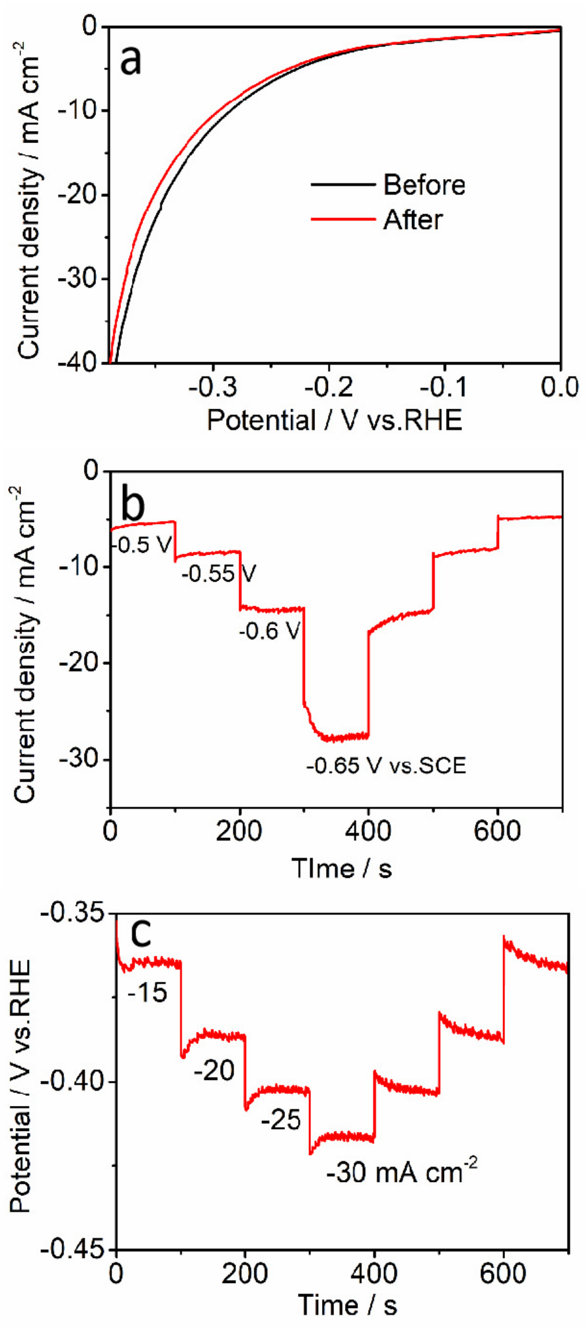

Figure S10. (a)the polarization curves of the electrode before and after 500 cycles. (b) the multi-potential steps and (c) the multi-current steps results.

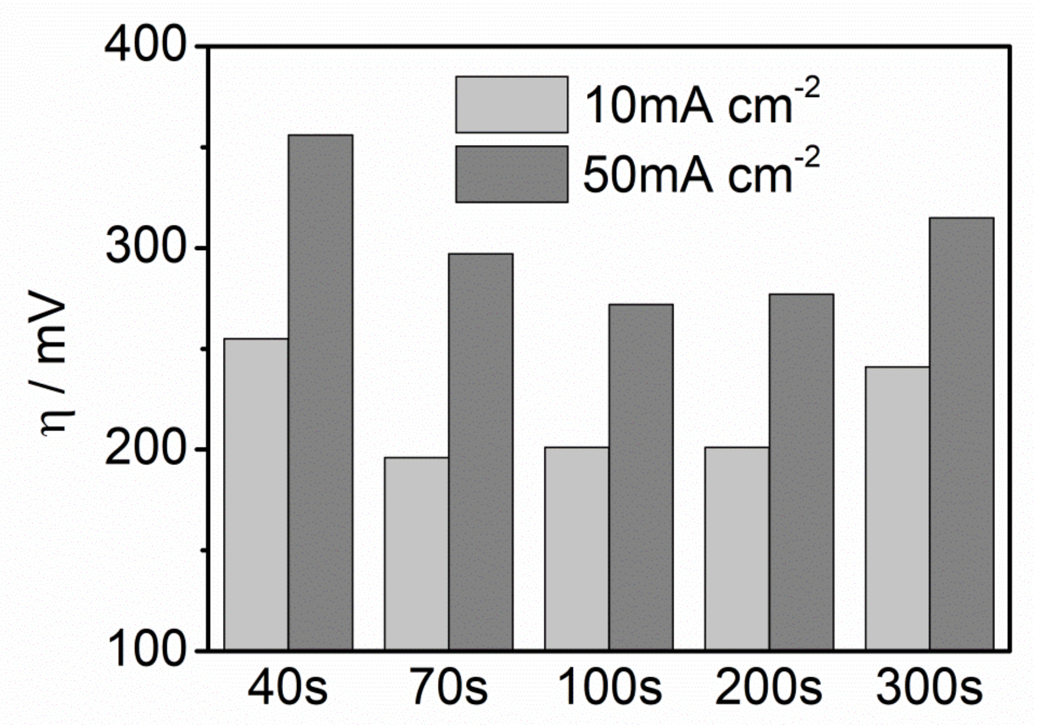

Figure S11. The comparison of the corresponding overpotentials at 10 mA cm^{-2} and 50 mA cm^{-2} current outputs based on different deposition time electrodes.

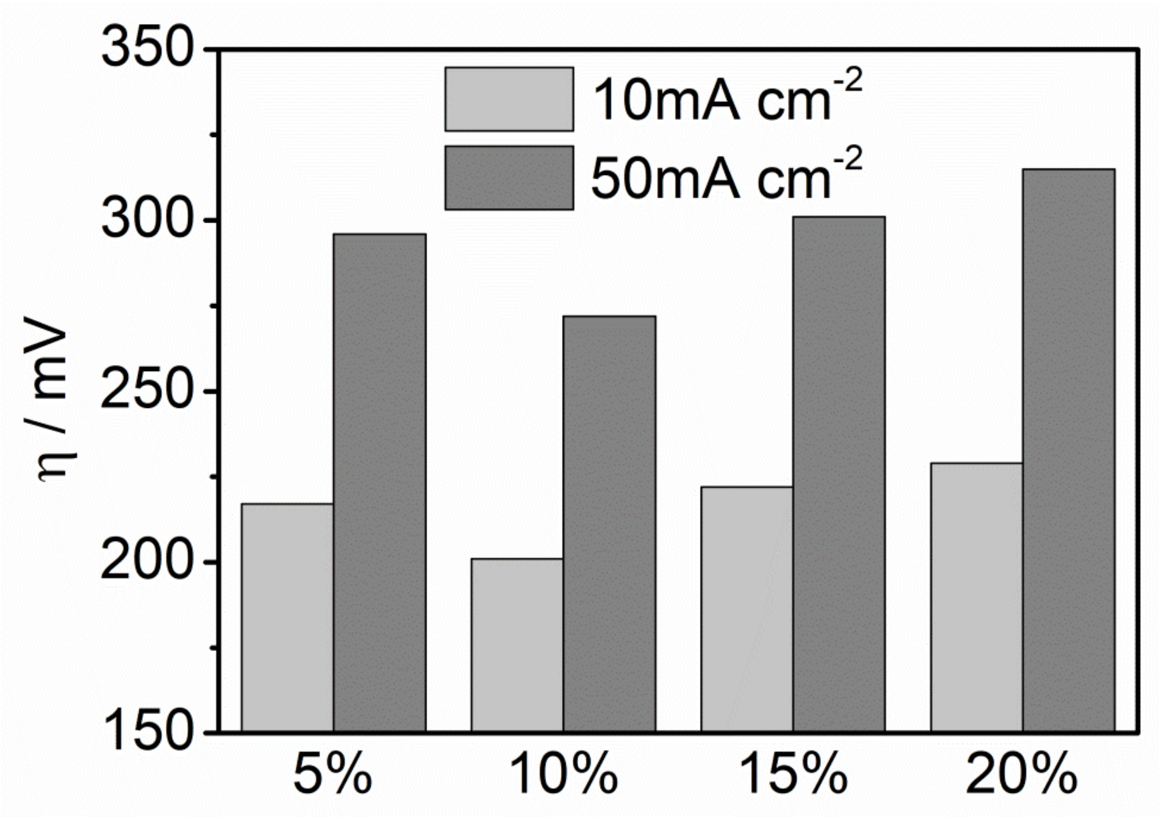

Figure S12. The comparison of the corresponding overpotentials at 10 mA cm^{-2} and 50 mA cm^{-2} current outputs based on different doping concentrations electrodes.

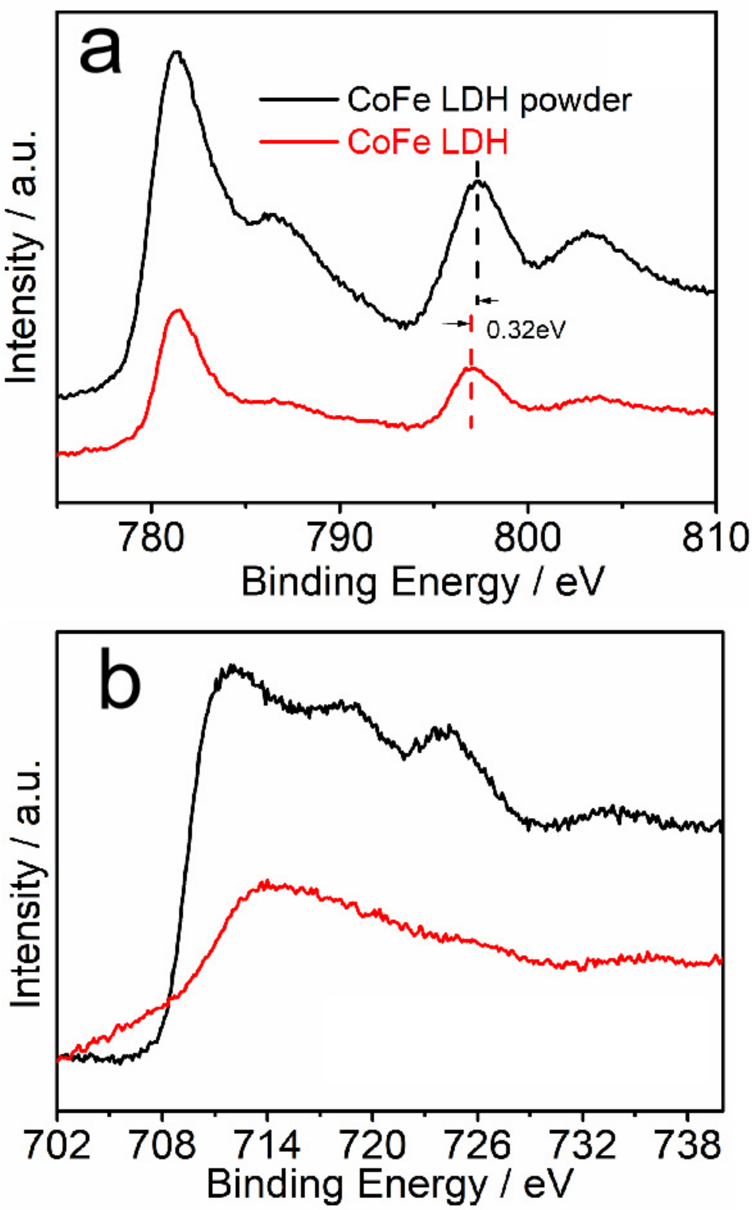

Figure.S13 The high-resolution (a) Co 2p and (b) Fe 2p spectra of CoFe LDH electrode and CoFe LDH powder. CoFe LDH powder sample was prepared according to our previously published article[16].

3. Supplementary Tables

Table S1. Summary of parameters for sample preparation.

Doping concentration	$Cr(NO_3)_3 \cdot 9H_2O$ (mmol)	$FeCl_2 \cdot 4H_2O$ (mmol)	$Co(NO_3)_2 \cdot 6H_2O$ (mmol)
0	0	1.25	
5%	0.1875	1.0625	
10%	0.375	0.875	2.5
15%	0.5625	0.6875	
20%	0.75	0.5	

Table S2. Comparison of the HER performance of CoFeCr LDH catalyst with other reported OER catalysts

Catalyst	Tafel slope (mV dec^{-1})	Overpotential (η/mV) Vs. 10mA cm^{-2}	Electrolyte	Reference
**Cr-CoFe LDH**	**105**	**201**	**1 M KOH**	**This work**
Co/CoP	73.8	253	1 M KOH	*Advanced Energy Materials, 2017, 7, 1602355.*
Mesoporous Thin-Film NiS2	N/A	250	1 M KOH	*ACS Catalysis, 2020, 10, 15114–15122.*
NiFe-LDH/NF	58.9	210	1 M KOH	*Science, 2014, 345, 1593–1596.*
1T-MoS_2	56	270		*Int. J. Hydrogen Energy, 2021,46, 8377-8390*
$Zn_{1-x}Fe_x$–LDH/NF	110	221	1 M KOH	*Small, 2018, 14, 1803638.*
EG/$Co_{0.85}$Se/NiFe-LDH	160	265	1 M KOH	*Energy Environ. Sci., 2016, 9, 478-483.*
Co_9S_8@NOSC	105	320	1 M KOH	*Advanced Science, 2018, 5, 1700464*
$Ni_{1-x}Fe_x$-LDH	110	242	1 M KOH	*ACS Applied Materials & Interfaces, 2018, 10, 42453−42468.*
$NdBaMn_2O_{5.5}$	87	290	1 M KOH	*ACS Catalysis, 2017, 8, 364-371.*
CoMoV LDH/NF	182	270	1 M KOH	*Chemical Communications, 2019, 55, 3521- 3524.*
NiFe-LDH NS/DG10	210	300	1 M KOH	*Advanced Materials, 2017, 29, 1700017.*
CoP@BCN	52	215	1 M KOH	*Advanced Science, 2018, 5, 1700464.*
CoFe LDH-F	95	255	1 M KOH	*ACS Applied Materials & Interfaces, 2016, 8, 34474−34481.*
CoFeNiMo@NCNT	67.4	209.9	0.5 M H_2SO_4	*Applied Catalysis B: Environmental, 2021, 280, 119421.*
NiFe-LDH/$FeCoS_2$/CFC	157	308	1 M KOH	*Journal of Materials Science, 2020, 55, 16625–16640.*
Ni/NiS	123.3	230	1 M KOH	*Advanced Functional Materials, 2016, 26, 3314–3323.*

4. Notes and references.

1. ZhongHua Xue, Hui Su, QiuYing Yu, Bing Zhang, HongHui Wang, XinHao Li, JieSheng Chen, Janus Co/CoP nanoparticles as efficient Mott-Schottky electrocatalysts for overall water splitting in wide pH range, *Advanced Energy Materials*, **2017**, 7, 1602355.
2. Cüneyt Karakaya, Navid Solati, Umut Savacı, Emre Keles, Servet Turan, Serdar C¸elebi, and Sarp Kay, Mesoporous thin-film NiS_2 as an idealized pre-electrocatalyst for a hydrogen evolution reaction. *ACS Catalysis*, **2020**, 10, 15114–15122.
3. Jingshan Luo, Jeong-Hyeok Im, Matthew T. Mayer, Marcel Schreier, Mohammad Khaja Nazeeruddin, Nam-Gyu Park, S. David Tilley,Hong Jin Fan, Michael Grätzel, Water photolysis at 12.3% efficiency via perovskite photovoltaics and Earth-abundant catalysts. *Science*, **2014**, 345, 1593–1596.
4. Selvaraj Venkateshwaran, Mukkath JosephJosline, Sakkarapalayam Murugesan Senthil Kumar, Fine-tuning interlayer spacing in MoS_2 forenriching 1T phase via alkylated ammonium ions for electrocatalytic hydrogen evolution reaction, *International Journal of Hydrogen Energy*, **2021**, 46, 8377-8390.
5. Gaddam Rajeshkhanna, Syam Kandula, Khem Raj Shrestha, Nam Hoon Kim, Joong Hee Lee, A new class of $Zn_{1-x}Fe_x$–oxyselenide and $Zn_{1-x}Fe_x$–LDH nanostructured material with remarkable bifunctional oxygen and hydrogen evolution electrocatalytic activities for overall water splitting, *Small*, **2018**, 14, 1803638.
6. Yang Hou, Martin R. Lohe, Jian Zhang, Shaohua Liu, Xiaodong Zhuang, Xinliang Feng, Vertically oriented cobalt selenide/NiFe layered-double-hydroxide nanosheets supported on exfoliated graphene foil: an efficient 3D electrode for overall water splitting, *Energy Environ. Sci.*, **2016**, 9, 478-483.
7. Nasir Mahmood, Yunduo Yao, Jing-Wen Zhang, Lun Pan, Xiangwen Zhang, Ji-Jun Zou, Electrocatalysts for hydrogen evolution in alkaline electrolytes:mechanisms, challenges, and prospective solutions, *Advanced Science*, **2018**, 5, 1700464.
8. Gaddam Rajeshkhanna, Thangjam Ibomcha Singh, Nam Hoon Kim, Joong-Hee Lee, Remarkable bifunctional oxygen and hydrogen evolution electrocatalytic activities with trace level Fe-doping in Ni- and Co-layered double hydroxides for overall water splitting. *ACS Applied Materials & Interfaces,* **2018**, 10, 42453−42468.
9. Jian Wang, Yang Gao, Dengjie Chen, Jiapeng Liu, Zhenbao Zhang, Zongping Shao, Francesco Ciucci, Water splitting with an enhanced bifunctional double perovskite. *ACS Catalysis*, **2017**, 8, 364-371.
10. Jian Bao, Zhaolong Wang, Junfeng Xie, Li Xu, Fengcai Lei, Meili Guan, Yan Zhao, Yunpeng Huang, Huaming Li, Ternary cobalt-molybdenum-vanadium layered double hydroxide nanosheet array as an efficient bifunctional electrocatalyst for overall water splitting. *Chemical Communications*, **2019**, 55, 3521- 3524.
11. Yi Jia, Longzhou Zhang, Guoping Gao, Hua Chen, Bei Wang, Jizhi Zhou, Mun Teng Soo, Min Hong, Xuecheng Yan, Guangren Qian, Jin Zou, Aijun Du, Xiangdong Yao, A heterostructure coupling of exfoliated Ni-Fe hydroxide nanosheet and defective graphene as a bifunctional electrocatalyst for overall water splitting. *Advanced Materials*, **2017**, 29, 1700017.
12. Peng Fei Liu, Shuang Yang, Bo Zhang, Hua Gui Yang, Defect-rich ultrathin cobalt-iron layered double hydroxide for electrochemical overall water splitting, *ACS Applied Materials & Interfaces*, **2016**, 8, 34474−34481.
13. Ting Wang, Ming Xu, Fengrui Li, Yunjiang Li, Weilin Chen, Multimetal-based nitrogen doped carbon nanotubes bifunctional electrocatalysts for triiodide reduction and water-splitting synthesized from polyoxometalate-intercalated layered double hydroxide pyrolysis strategy, *Applied Catalysis B: Environmental*, **2021**, 280, 119421.
14. Zewu Zhang, Jiamin Zhou , Hanlin Wei, Yifan Dai, Shijia Li, Haojun Shi, Gang Xu, Construction of hierarchical NiFe-LDH/$FeCoS_2$/CFC composites as efficient bifunctional electrocatalysts for hydrogen and oxygen evolution reaction, *Journal of Materials Science*, **2020**, 55, 16625–16640.
15. Gao-Feng Chen, Tian Yi Ma, Zhao-Qing Liu, Nan Li , Yu-Zhi Su, Kenneth Davey, Shi-Zhang Qiao, Efficient and stable bifunctional electrocatalysts Ni/Ni_xM_y(M = P, S) for overall water splitting, *Advanced Functional Materials*, **2016**, 26, 3314–3323.
16. M. Y. Li, J. J. Zhang, X. Li, W. W. Bao, C. M. Yang, C. Q. Jin, M. Li, S. M. Wang and N. N. Zhang, Tuning the electronic structures of self-supported vertically aligned CoFe LDH arrays integrated with Ni foam toward highly efficient electrocatalytic water oxidation, *New J. Chem.*, **2021**, 45, 13266-13270.
